# Resistance and Recovery of Methane-Oxidizing Communities Depends on Stress Regime and History; A Microcosm Study

**DOI:** 10.3389/fmicb.2018.01714

**Published:** 2018-07-31

**Authors:** Henri van Kruistum, Paul L. E. Bodelier, Adrian Ho, Marion Meima-Franke, Annelies J. Veraart

**Affiliations:** ^1^Department Microbial Ecology, Netherlands Institute of Ecology (NIOO-KNAW), Wageningen, Netherlands; ^2^Institut für Mikrobiologie, Leibniz Universität Hannover, Hanover, Germany; ^3^Department Aquatic Ecology and Environmental Biology, Institute for Water and Wetland Research, Radboud University, Nijmegen, Netherlands

**Keywords:** ammonium toxicity, soil drying, ecosystem functioning, methanotrophy, methane oxidation, resilience, soil microbiome

## Abstract

Although soil microbes are responsible for important ecosystem functions, and soils are under increasing environmental pressure, little is known about their resistance and resilience to multiple stressors. Here, we test resistance and recovery of soil methane-oxidizing communities to two different, repeated, perturbations: soil drying, ammonium addition and their combination. In replicated soil microcosms we measured methane oxidation before and after perturbations, while monitoring microbial abundance and community composition using quantitative PCR assays for the bacterial 16S rRNA and *pmoA* gene, and sequencing of the bacterial 16S rRNA gene. Although microbial community composition changed after soil drying, methane oxidation rates recovered, even after four desiccation events. Moreover, microcosms subjected to soil drying recovered significantly better from ammonium addition compared to microcosms not subjected to soil drying. Our results show the flexibility of microbial communities, even if abundances of dominant populations drop, ecosystem functions can recover. In addition, a history of stress may induce changes in community composition and functioning, which may in turn affect its future tolerance to different stressors.

## Introduction

Soils and their microbial communities are under increasing environmental pressure. Climate change will lead to longer, and more frequent periods of drought in large areas around the globe (Stocker et al., 2013). In addition, many soils are facing effects of increased atmospheric nitrogen deposition and over-fertilization, as well as other stressors such as rising temperature, salinization, compaction, and pollution (Galloway et al., 2004; Smith et al., 2016). Because soil microbial communities deliver a wide range of ecosystem services, there is an increased interest in the resistance and resilience of microbial communities and the functions they mediate (De Vries et al., 2012; Griffiths and Philippot, 2013). Indeed, microbial resistance and resilience to single stress events (Wertz et al., 2007; Wittebolle et al., 2009; Ho et al., 2011), recurring stress events (Ho et al., 2016c; Jurburg et al., 2017), and the role of site history in conferring resilience to the contemporary community (Ho et al., 2016b) have recently been investigated. However, soil microbial communities face recurring perturbations of different magnitude and nature, which may shape their community composition, alter their future resilience, and affect their critical functions.

Because microbial communities contain distinct species carrying out the same function, but responding differently to a changing environment, it may be hard to capture a change in broad function as a response to disturbance or stress. Soil functions for which a large functional redundancy is assumed, like respiration, are less likely to be affected by perturbations compared to more specific functions, executed by a narrower group of microbes (Griffiths and Philippot, 2013). Therefore, it has been proposed that the effect of stress on a soil ecosystem can better be determined by measuring a specific soil function (Ho et al., 2016c). Here, we focus on aerobic methane oxidation, a well-defined process executed by a specific group of microorganisms: the methane-oxidizing bacteria (MOB) (Ho et al., 2013). Methane oxidation by MOB is the largest biological sink of the greenhouse gas methane, and thus an environmentally relevant soil function.

All currently known aerobic MOB are contained into three microbial phyla: *Proteobacteria, Verrucomicrobia*, and the candidate phylum NC10 involved in methane oxidation coupled to denitrification. Based on this phylogeny, aerobic MOB have been categorized into three types (Knief, 2015). Type I MOB are members of the *Gammaproteobacteria*, while type II MOB belong to the *Alphaproteobacteria*, and the third type belong to the phylum *Verrucomicrobia*. Although members of NC10 have been detected in terrestrial ecosystems (e.g., soils), their relative contribution to *in-situ* methane oxidation is less known or appeared to be marginal (Blazewicz et al., 2012). The vast majority of active MOB in soil ecosystems belong to the *Proteobacteria* (Ho et al., 2013).

Ecosystem responses to stress can be defined as their resistance, i.e., the ability to withstand a perturbation without loss of biomass or functionality, and their resilience. The resilience of a system can be roughly defined as either the ability to recover from stress, also referred to as “engineering resilience,” or the potential to remain in a dynamic equilibrium when exposed to stress, without tipping into another, alternative equilibrium, referred to as “ecological resilience” (Holling, 1973; Scheffer et al., 2001; Griffiths and Philippot, 2013). Here, by “resilience” we mean the ability to recover from stress.

Earlier work on stress resistance of MOB focused mainly on the effects of desiccation and ammonium stress. MOB communities do not resist drought, but show a remarkable resilience to desiccation, even after long-term drought. This has been attributed to the large amount of desiccation-resistant exospores some MOB are known to produce (Whittenbury et al., 1970; Ho et al., 2011; Collet et al., 2015). However, methane oxidation rates decreased with increasing intensity of recurring desiccation-rewetting events, suggesting that this resilience is not infinite (Ho et al., 2016c). In addition, soil history is of importance, and has been shown to exert a more pronounced effect on the MOB community composition than activity (Ho et al., 2016b). Stress shapes soil microbial communities, selecting for those harboring the right trait combination to endure stress. This environmental “legacy” defines future ecological resilience to stress of the same nature, but may trade-off into yet undiscovered changes in community function.

The response of MOB to ammonium addition is more complex. On one hand, ammonium is a competitive inhibitor of the particulate methane monooxygenase (pMMO), the key enzyme for aerobic methane oxidation. This is due to the fact that ammonia monooxygenase (AMO) and pMMO are evolutionary related, and use similar substrates (Holmes et al., 1995). Therefore, pMMO can also oxidize ammonium, and vice versa (Bodelier and Frenzel, 1999). Numerous studies have shown that this interaction can inhibit methane oxidation by MOB, especially when the methane concentration is low (Schnell and King, 1994; Gulledge and Schimel, 1998; Nold et al., 1999). A study by Nyerges and Stein (2009) shows that the methane to ammonium ratio is critical for the extent of this effect, confirming the competitive nature of ammonium inhibition. On the other hand, ammonium addition has also been shown to increase methane oxidation by MOB, especially in nitrogen-limited soils (De Visscher et al., 1999; Bodelier et al., 2000; De Visscher and Van Cleemput, 2003). This phenomenon has been linked to the high nitrogen requirement of MOB. While some MOB are capable of nitrogen fixation, this is a costly process requiring a lot of energy and reducing equivalents. The presence of ammonium has therefore been hypothesized to boost growth of MOB by relieving this nitrogen pressure (Bodelier and Laanbroek, 2004).

Some studies show that although MOB in soil regained activity after a disturbance, the microbial composition changed (Ho et al., 2011, 2016c). There is evidence that these changes in microbial composition due to a history of stress have an effect on the resistance to other types of stress (e.g., communities tolerant to salt stress also appear to be more resistant to desiccation (Baumann and Marschner, 2013). Moreover, a review by Azarbad et al. (2016) summarizes stress-on-stress studies regarding metal toxicity. They conclude that microbes that have acquired resistance mechanisms to metal poisoning have an increased resistance against some related stresses, but a decreased resistance against other stresses. This suggests that stress resistance can come at a price: acquiring a trait, such as resistance against a certain stressor, costs energy, and diverts resources from other traits. Therefore, the community may be less resilient when facing other stresses. At the same time, stress-on-stress can also increase resilience to further disturbances by selecting for more adaptive or stress-resilient community members (Griffiths et al., 2001; Tobor-Kapłon et al., 2006). Here, we further explore effects of multiple stressors on methanotrophic communities.

**FIGURE 1:**
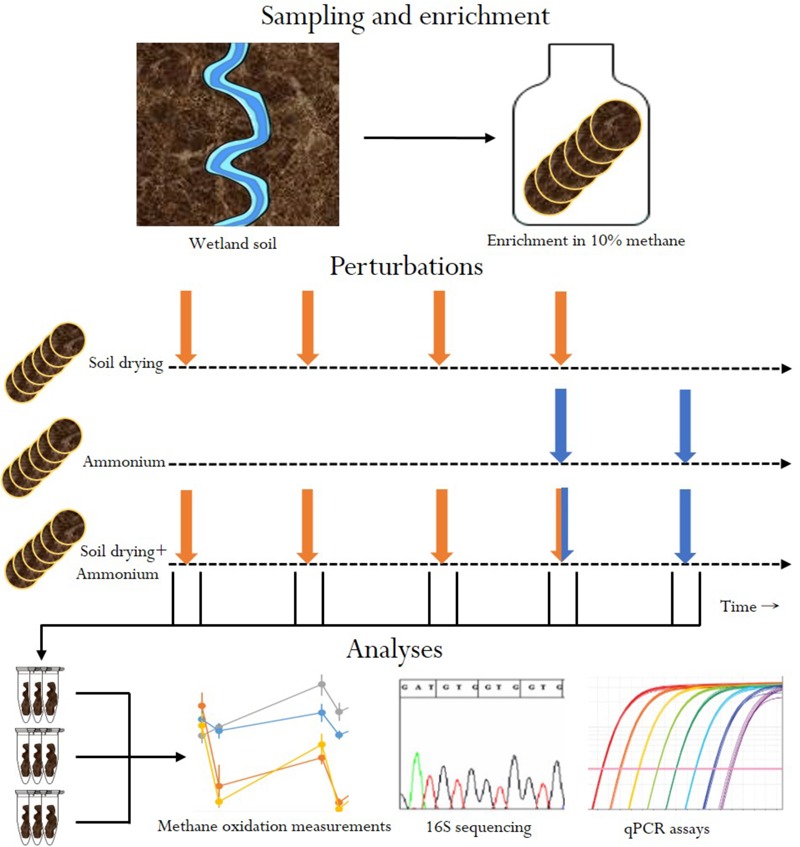
Overview of experimental design. Orange arrows indicate soil drying events. Blue arrows indicate ammonium addition.

In this *in vitro* experiment, we tested how stress-on-stress affects MOB community composition and functioning, by subjecting soils enriched in methanotroph abundance to four (weekly) soil drying events, followed by two ammonium applications. We followed community composition and recovery of (*in vitro*) methane oxidation rates before and after perturbation in soil-dried microcosms, ammonium microcosms and stress-on-stress soil-dried^∗^ammonium microcosms (**Figure 1**).

## Results

### Methane Oxidation Rates

**FIGURE 2:**
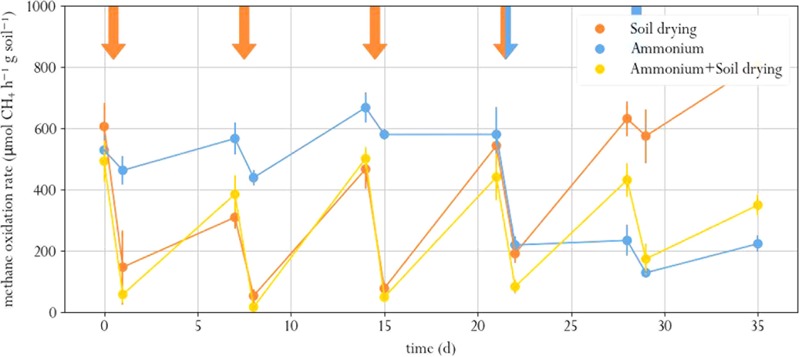
Methane oxidation rate in soil microcosms exposed to different perturbation regimes. Orange arrows indicate soil drying, blue arrows indicate ammonium addition to the corrensponding treatments. Error bars represent standard error of the mean.

Soil drying stress resulted in significantly lowered methane oxidation rates, as compared to the pre-perturbation (t_0_) samples (Paired *T* test, p.adj < 0.02 for all cases, **Figure 2**, and Supplementary Table S1.1). After the first perturbation, in 1 week, activity partially recovered to roughly half the t_0_ level. Subsequent perturbations did not lower the methane oxidation rate any further. In fact, the microcosms exposed to only soil drying had similar methane oxidation rates at the last three timepoints. In a previous study, a one-off and recurring perturbation resulted in a full recovery, but increasing the intensity of recurring desiccation led to decreased methane oxidation rate in the long term (Ho and Frenzel, 2012; Ho et al., 2016b).

Ammonium addition also resulted in lower methane oxidation rate compared to the t_0_ samples, both in the microcosms amended with ammonium as sole treatment and in the microcosms that were also exposed to soil drying (p.adj < 0.01 for both cases, **Figure 2**). This indicates that the used ammonium concentration was indeed inhibitory. However, only the microcosms that were exposed to soil drying prior to ammonium addition partially recovered from this perturbation. One week after ammonium addition, these showed a significantly higher methane oxidation rate compared to those microcosms that only underwent ammonium-stress (day 28 and 35). The microcosms that were only exposed to ammonium addition showed no significant recovery.

### Methanotroph Abundance

**FIGURE 3:**
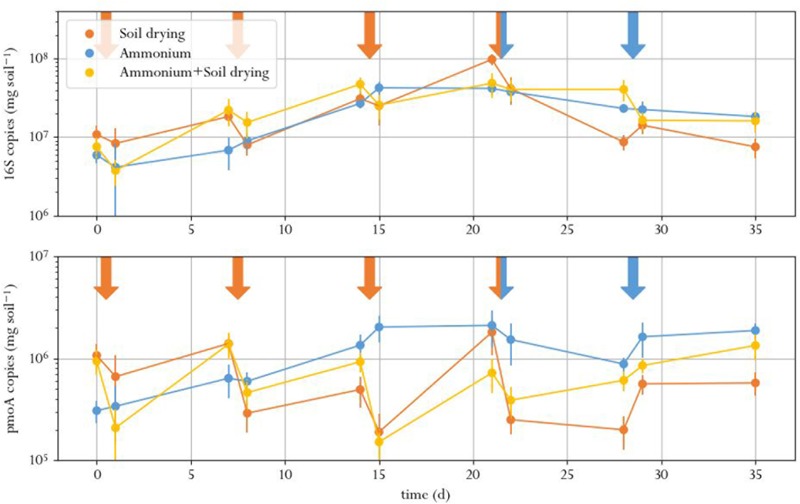
Total amount of 16S (top) and *pmoA* (bottom) copies in soil microcosms, exposed to different perturbation regimes. Orange arrows indicate soil drying events, blue arrows indicate ammonium addition to the corresponding treatment. Error bars represent standard error of the mean.

The number of 16S rRNA gene copies, indicating total bacterial abundance, did not signficantly differ between treatments at the same timepoint for all time points except t9 (**Figure 3** and Supplementary Table S1.2). Instead, the amount of 16S copies showed an increasing trend for all treatments in the first half of the experiment, after which it leveled off. This was expected, as earlier studies that performed 16S qPCR on perturbed soil samples did not find a treatment effect as well (Ho et al., 2011, 2016c; Barnard et al., 2013). This result underlines the need for monitoring specific functions when evaluating soil function resilience as a result of stress: in conditions where some species struggle, others thrive and can take their place. This is especially true for soils subjected to soil drying, in which surviving cells can grow swiftly on the organic matter released by cell lysates of desiccation-sensitive species (Bottner, 1985; Van Gestel et al., 1993). This results in a poor correlation between total bacterial abundance in soil and soil function.

The number of *pmoA* copies, indicating MOB abundance, in the microcosms dropped after every soil drying treatment in the corresponding microcosms, as compared to the same samples before the treatment (**Figure 3**). However, a significant effect of soil drying on *pmoA* abundance was only found at day 14, 15, 21, 28, 29, and 35 (Supplementary Table S1.3 and Supplementary Figure S1).

Copy numbers of *pmoA* did not significantly correlate to methane oxidation rates. Although both the number of *pmoA* copies as well as the methane oxidation activity dropped after soil drying, the difference in *pmoA* copy numbers between treatments was not as pronounced as the difference in methane oxidation rates. Moreover, *pmoA* copy numbers after soil drying did not show the same recovery compared to the methane oxidation rate toward the end of the experiment. This indicated that this recovery may not be due to an increase of MOB in numbers, but rather due to increased methane oxidation per cell, or a shift in microbial composition toward more active types of MOB.

### Microbial Community Composition

Recent findings show that not only MOB, but also accompanying microorganisms are relevant in modulating methane oxidation (Ho et al., 2016a; Veraart et al., 2018). Therefore, we analyzed changes in microbial community composition by sequencing the 16S rRNA gene, and considered both changes in methanotrophs and non-methanotrophs.

**FIGURE 4:**
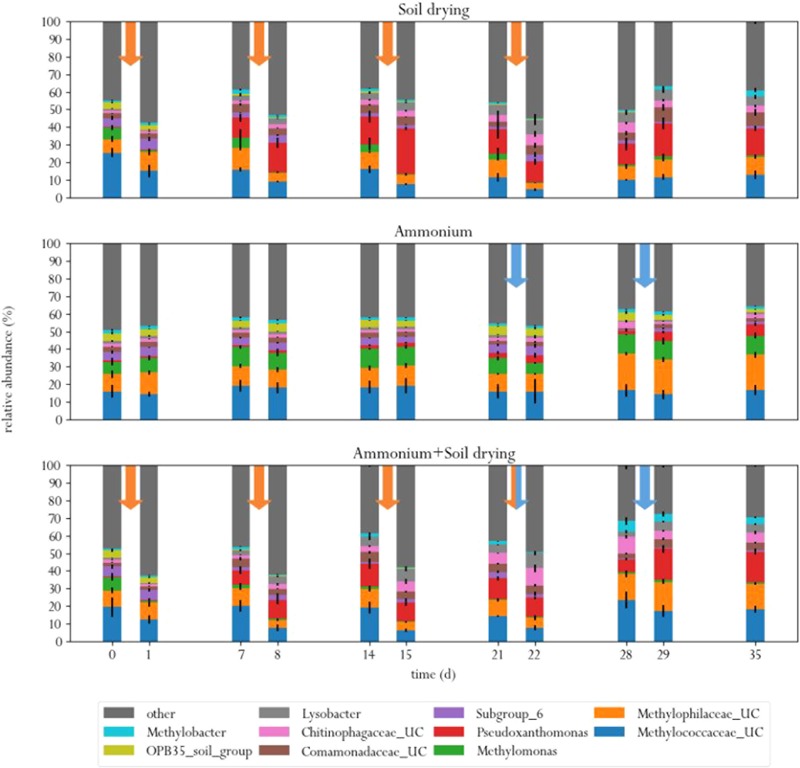
Relative abundances of ten most abundant bacterial genera in the three different treatments, before and after each perturbation. Orange arrows indicate soil drying events, blue arrows indicate ammonium addition to the corrensponding treatments.

**FIGURE 5:**
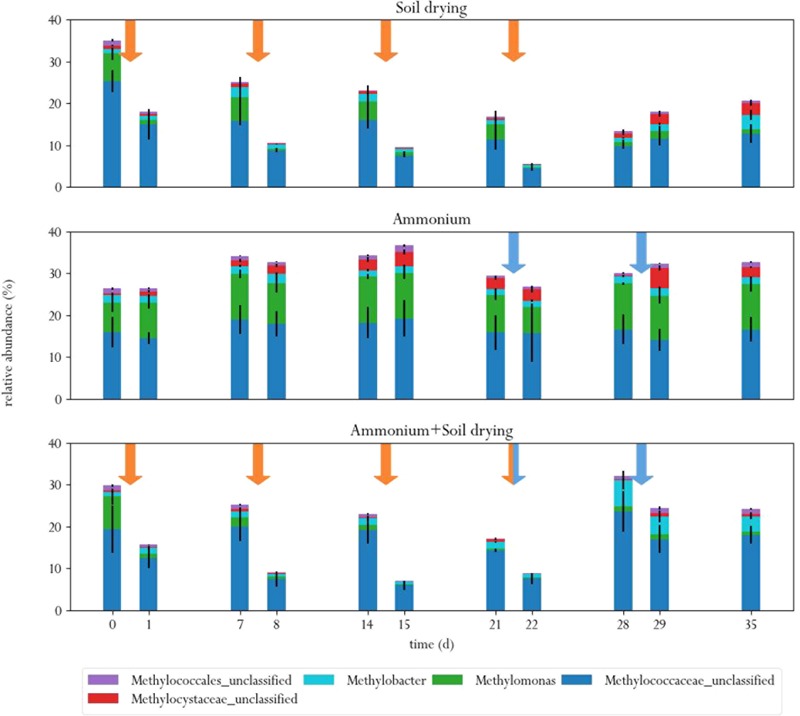
Relative abundances of MOB in the three different treatments, before and after each perturbation. Orange arrows indicate soil drying events, blue arrows indicate ammonium addition to the corrensponding treatments.

At the start of the experiment, three genera were the predominant methanotrophs in all samples: *Methylomonas*, an unclassified genus from the family *Methylococcaceae* (99% related to *Methylomonas paludis* strain MG30) and an unclassified genus from the family *Methylophilaceae* (**Figure 4**). The first two are type I MOB, the last genus is a group of methylotrophs, which are often observed to co-occur with MOB, as they can grow on methanol produced by the MOB (McDonald and Murrell, 1997; Krause et al., 2017). This is a logical consequence of the enrichment procedure prior to the experiment, where soil microcosms were placed in a 10% methane atmosphere, giving MOB a selective advantage. These groups of bacteria also dominated in a study on methane oxidation coupled to denitrification in a bioreactor inocculated with landfill sediments (Liu et al., 2014). In the control group, these three genera remained the predominant phylogenetic groups in the soil across the entire experiment. Noteworthy, *Methylomonas* was already more abundant in the control group than in other treatment groups, at the start of the experiment. Type II MOB were less abundant than type I MOB in the microcosm soils, covering about 0.5–3% of the total microbial population, depending on the sample (**Figure 5**).

Soil drying caused major shifts in the microbial community. Both dominant groups of MOB decreased in relative abundance after treatment. This effect was especially evident for *Methylomonas*, which was barely present after a few dessication cycles. The population of *Methylococcaceae* recovered at the end of the experiment, but *Methylomonas* stayed absent until the end of the experiment. In addition, an increase in MOB from the genus *Methylobacter* was observed at the end of the experiment. Some other groups of bacteria also increased in relative abundance after soil drying, most notably from the genus *Pseudoxanthomonas*, but also bacteria from the families *Chitinophagaceae* and *Comamonadaceae* and the genus *Lysobacter* showed an increase in relative abundance. Bacteria from the genus *Pseudoxanthomonas* have earlier been observed in co-occurrence with MOB, and is associated with denitrification (Liu et al., 2014; Karthikeyan et al., 2015). *Lysobacter* is a notable polymer degrader (McKay and Donaghy, 1995), and its exclusive presence in soil samples that have been desiccated suggests that this genus feasts on the remains of cells lysed by osmotic stress.

In the ammonium-treatment microcosms, ammonium addition resulted in an increase in relative abundance of bacteria from the family *Methylophilaceae*, after a 1 week delay. Besides this change, the microbial composition remained relatively similar compared to the control group. The drop in methane oxidation rates after ammonium addition, along with the fact that changes in microbial composition after ammonium addition in this group are fairly minor suggests that ammonium is an inhibitor of pMMO, but does not have a bacteriocidal effect at the applied concentration.

The microcosms that received stress-on-stress of both soil drying and ammonium perturbations showed the same response to soil drying as the dried-only microcosms, but had a different response to ammonium addition compared to the microcosms receiving only ammonium. The soil-dried^∗^ammonium microcosms showed the same relative increase in bacteria from the family *Methylophilaceae*, but also a relative increase in MOB from the *Methylococcaceae* family and *Methylobacter* genus. These MOB may be responsible for the partial recovery in methane oxidation rate in the soil-dried^∗^ammonium microcosms 1 week after ammonium addition.

**FIGURE 6:**
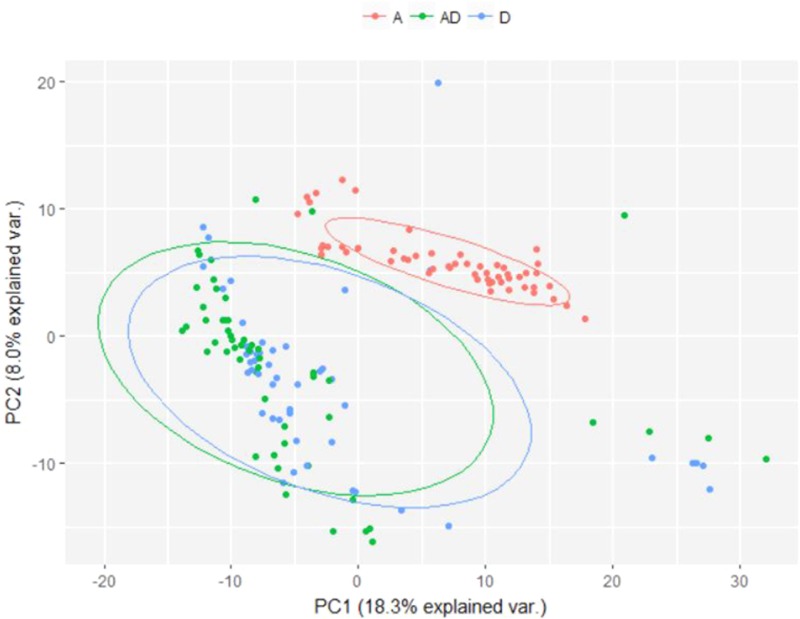
PCA plot showing differences in microbial composition between soil samples subjected to different perturbation regimes. Samples from day 0 were excluded, as no treatment was applied yet. A, Ammonium; D, Soil drying.

The difference in microbial composition between soil samples exposed to soil drying and those that were not was visualized using Principal Component Analysis (PCA) (**Figure 6**), which shows a clear distinction between samples subjected to soil drying and the other samples. This effect is not observed for ammonium addition.

### Resistance and Recovery

**FIGURE 7:**
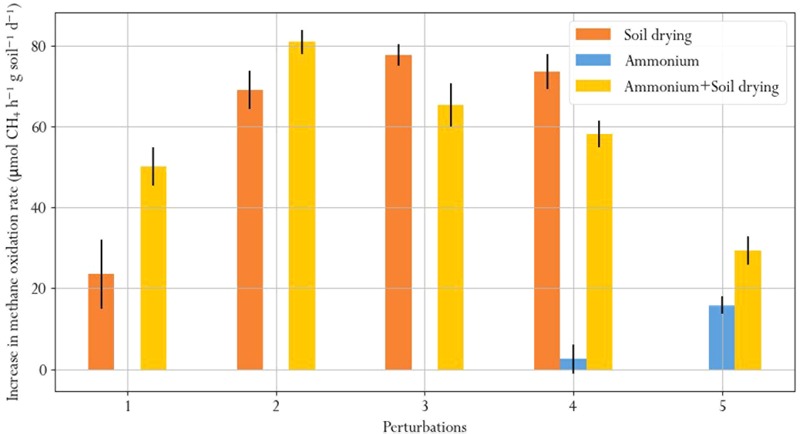
Average daily increase in methane oxidation rate during the first 6 days after each perturbation, indicating recovery.

We quantified recovery after soil drying and ammonium perturbations, from the change in methane oxidation rate after disturbance in the first 6 days after each perturbation (**Figure 7**). Recovery from soil drying increased after the second perturbation, but subsequent perturbations did not further increase the recovery rate. Microcosms subjected to ammonium-only perturbations barely recovered, as shown from appreciably small changes in the methane oxidation rate. However, recovery of methane oxidation after ammonium addition was significantly higher in the soil-dried^∗^ammonium samples, which had already been subjected to soil drying. This suggests that soil drying modifies the resilience of the microbial community to ammonium addition perturbations.

## Discussion

### Recovery After Soil Drying

In this study, we tested resistance and resilience of methane-oxidizing communities, to better understand how environmental stress affects the methane oxidation potential of soils. We tested resistance by subjecting soil microcosms to four soil drying perturbations, two ammonium perturbations, or both, and inferred resilience from the recovery of methane oxidation potential and MOB community composition over time. In the soil microcosms, methane oxidation rate decreased significantly after drought events, but rates recovered within days. Recovery rates increased after a second soil drying perturbation, and stabilized after this point. Interestingly, although soil drying hardly affected methane oxidation rates after a few days of recovery, the microbial community composition shifted, affecting the relative abundances of methanotrophs and non-methanotrophs. The methane oxidation potential was neither resistant nor resilient to ammonium stress, except for those soils that underwent both soil drying perturbations and ammonium addition. This indicates that there is no generic stress-response of microbial communities, as previously postulated by Griffiths and Philippot (2013), but soil stability can be probed by perturbations of the expected nature.

### Ammonium Inhibition: Stress-on-Stress Versus Single Stress Effects

A possible explanation for the better recovery of MOB communities subjected to stress-on-stress, lies in the emergence of *Methylobacter* species after ammonium addition in the microcosms receiving ammonium and soil drying perturbations, but not in the microcosms that only received ammonium. Soil drying perturbations prior to ammonium addition decreased the amount of MOB by ∼60%, opening up a niche for different species of MOB to recolonize. Ammonium addition stimulated growth of MOB in microcosms after soil drying, which is clearly visible in the difference in relative abundance of MOB between the soil drying only and soil-dried^∗^ammonium treatments 1 week after ammonium addition (day 28). The emergence of type Ia MOB, including *Methylobacter* is consistent with their anticipated life strategy as ruderals, capitalizing on the sudden availability of ammonium and other nutrients from cell die-off, under a high methane availability (Ho et al., 2013). Similarly, *Methylobacter*, have been found to increase in abundance after soil drying and rewetting in other studies, indicating desiccation resistance even without the ability to produce hardy resting cells (Bodelier et al., 2013; Pan et al., 2014; Collet et al., 2015; Ho et al., 2016c). Additionally, an unclassified genus from the family *Methylococcaceae* increased in abundance drastically in the soil-dried^∗^ammonium treatment after ammonium addition. This may be a result of recovery from the soil drying treatment, as such increase was observed a week after every soil drying perturbation, albeit less pronounced.

However, this does not explain how the microcosms in the ammonium and soil drying treatment could overcome ammonium inhibition of pMMO, while the microcosms subjected to only ammonium could not. The lack of change in the microbial composition of the ammonium treatment group after ammonium addition suggests that ammonium addition did not have a bacteriocidal effect, but rather inhibited pMMO.

There are several possible explanations for the induced ammonium resistance. One explanation may lie in the nitrogen metabolism of the total microbial community. After soil drying events we observed an increase in phylogenetic groups associated to denitrification, including members of the genus *Pseudoxanthomonas*, and *Chitinophagaceae* and *Comamonadaceae* families (Finkmann et al., 2000; Khan et al., 2002; Thierry et al., 2004). In addition, MOB have several ways to deal with excess nitrogen, including denitrification pathways, for example present in some *Methylomonas, Methylomicrobium*, and *Methylocystis* species (Stein and Klotz, 2011; Kits et al., 2015a,b). The presence of MOB and non-MOB denitrifiers could have contributed to lowering the nitrogen content in the soil after ammonium addition: MOB convert ammonium into nitrite (we did not observe ammonium oxidizers), which is further converted to nitrous oxide or nitrogen gas by denitrifying MOB or canonical denitrifiers present in anaerobic aggregates.

Another explanation could be that soil drying selects for MOB species that adopt more resilient life-history strategies. In our experiment, soil drying may have selected for stress-tolerators and ruderals, rather than competitors (Ho et al., 2013; Krause et al., 2014). The trait-pool resulting from four soil drying events may have been not only suited to survive soil drying, but also more suited to overcome ammonium inhibition. Moreover, our findings can be due to legacy effects (Ho et al., 2016b), as our soils originated from river floodplain are subjected to frequent drying and flooding cycles, but not to regular ammonium fertilization, withstanding the first type of stress but not the latter.

### Limitations and Ecological Context

In this study, soil from a natural system was transferred to a laboratory setup, and subsequently exposed to two distinct stressors. The advantage of this setup is that it reduces the noise present in natural systems, allowing us to quantify the effect of perturbations on the microbial community in this soil more precisely. However, this approach does not capture the full complexity of natural systems. For instance, the “batch effect” we encountered when setting up the microcosms shows a level of heterogeneity in the natural soil ecosystem that was not captured by our microcosms. Also, soils in natural systems are subject to more complex and irregular combinations of stressors compared to the approach taken here. Additionally, the outcome of such a laboratory experiment may be partially dependent on the ecosystem the soil was sampled from, as a history of stress has its influence on the microbes’ ability to withstand subsequent perturbations (Ho et al., 2016b; Krause et al., 2018). Although lab experiments such as this study are essential to disentangle microbial community dynamics, follow-up research on natural soils is necessary to verify these results in a broader ecological context.

## Conclusion

Repeated soil drying events changed the microbial community of floodplain soils, while restoring methane oxidation rates after a few days of recovery. Recovery rates of methane oxidation increased after repeated soil drying perturbations, showing adaptive potential of the microbial community. The reaction of the community depends on the type of stress: communities were neither resistant nor resilient to ammonium addition, unless they had a history of drought stress. In addition, our results suggest that stress responses depend on the life strategies of the MOB present, suggesting the importance of microbial eco-physiological knowledge when predicting impact of climate change on greenhouse gas emissions.

## Materials and Methods

### Experimental Design

In this experiment, MOB were subjected to two distinct types of perturbations: soil drying and ammonium addition. First, a soil sample was enriched for MOB by pre-incubating the soil in an airtight jar at 10% (v/v) methane for 3 weeks. This enrichment step was performed to ensure detection of MOB at high enough numbers to detect community changes using 16S sequencing. After enrichment, four soil treatments were set up simultaneously from this initial batch of soil: (1) six control microcosms, to which no stress was applied, (2) six microcosms only treated with soil drying, (3) six microcosms only treated with ammonium, (4) and finally six microcosms receiving stress-on-stress of both perturbations (**Figure 1**). Methane oxidation rates of all soil samples were measured before and after perturbations, to measure stress resistance and recovery. Dessication stress was applied four times, at 1-week intervals. Ammonium addition was performed twice, at 1-week intervals, starting at the fourth soil drying perturbation.

Unfortunately, when setting up the control microcosms the soil was not properly mixed, leading to a different initial starting community composition in the t_0_ control microcosms, whereas communities in the treatment microcosms were similar (See Supplementary Figures S2–S4 for control community dynamics, and Supplementary Figure S5 for PCoA analysis of all samples). Because the set-up of the experiment, sampling before and after perturbation still allowed for comparisons between groups, these data are shown in the manuscript.

### Soil Sampling and Microcosms

We collected the top layer (10 cm) of soil from a regularly flooded wetland next to the river Waal, the Netherlands (N 51° 52′ E 05° 53′), using a manual drill. The physico-chemical properties of this soil are well characterized (Bodelier et al., 2012), and this soil is used in several other studies (Steenbergh et al., 2009; Bodelier et al., 2012, 2013; Krause et al., 2013). The site is hydrologically isolated, only influenced by the river Waal. The top soil consists of sandy loam, changing to silt-loam at 20 cm depth, and is covered by *Lolium perenne*. Flooding occurs several weeks per year. Changes in atmospheric methane concentration have been attributed to wetlands, making this soil type particularly interesting for research regarding MOB (Bousquet et al., 2006). The collected soil was sieved (2 mm), homogenized, and pre-incubated at 10% (v/v) methane for 3 weeks before starting the experiment. After pre-incubation, we set up microcosms by dividing the soil over 24 petri dishes, six replicates per treatment. These were incubated in an airtight jar, at 10% (v/v) methane. Soil moisture content was kept at 35% (w/w) throughout the experiment (except for soil drying events) by weekly weighing the microcosms and replenishing evaporated water.

### Soil Drying and Ammonium Perturbations

For the soil drying perturbations, we left the opened microcosms in a laminar flow cabinet overnight (Clean Air ES/FB, Telstar Life Science Solutions, Utrecht, Netherlands), which resulted in ∼95% gravimetric water loss. After desiccation, but before methane oxidation measurements, evaporated water was replenished by adding the corresponding amount of demineralized water after weighing the microcosm. Previously, methanotrophic activity and community composition were shown to be resilient to drought events ranging from 5 to 22 years; the recovering activity and active community members were comparable in soils experiencing drought for <9 years, suggesting that the methanotrophs were more responsive to the induction of drought-rewetting, rather than the length of the drought event up to 9 years (Collet et al., 2015). Given the high methane concentration in which our samples were incubated and the competitive nature of pMMO inhibition by ammonium, we had to add a high amount of ammonium as well in order to inhibit pMMO. Ammonium was added to raise its concentration in the pore water to 50 mM, by spraying a 3 M solution of NH_4_Cl_2_ onto the soils, according to the following formula:

$$V\;=\;W_{\mathrm{soil}}*\mu *\frac{C_{ammonium-desired}}{C_{ammonium-stock}}$$

Where _W_soil__ represents the weight of the soil (g), _μ_ is the gravimetric water content of the soil, and _C_ammonium-desired__ and _C_ammonium-stock__ represent the concentration (M) of ammonium in the soil porewater after addition and in the stock solution, respectively. This ammonium concentration was expected to be inhibiting for pMMO, based on earlier studies on the effect of the methane/ammonium ratio on methane oxidation rate (Nyerges and Stein, 2009). Because the layer of soil in the microcosm was relatively thin (∼0.5 cm), and the ammonium solution was sprayed on the surface evenly, we assumed that the ammonium would diffuse evenly throughout the soil after addition.

### Methane Oxidation Rates

Methane oxidation rates were measured in 0.5 g soil sub-samples. To standardize CH_4_ and O_2_ diffusion, but maintain soil structure, each 0.5 g soil sample was put into a small half-open plastic container constructed from a cut Eppendorf tube (1.5 ml), after which the sample and container were put into an airtight tube (Exetainer, Labco, United Kingdom). This way we ensured each soil sub-sample had the same weight and shape, minimizing variation in diffusion. Subsequently, we added 1% (v/v) methane, and measured methane concentrations every 2 h for 4 h, by injecting 0.25 ml of sample into a an Ultra GC gas chromatograph (Interscience, Breda, Netherlands), equipped with a flame ionization detector (FID) and an Rt-Q-Bond (30 m, 0.32 mm, ID) capillary column. The decrease in methane concentration was linear for almost all measurements (*R*_2_ > 0.95). Non-linear measurements (for instance due to a leaking tube) were discarded before calculating methane oxidation rates. A control tube without soil was added for each set of measurements. Methane oxidation rate was the calculated according to the following formula:

$$\;v_{ch4ox}=\;\frac{{m*V}_{\mathrm{chamber}}}{W_{\mathrm{sample}}}*\frac{1E6}{m_{CH4}}*(1-\mu )$$

Where v_ch4ox_ is the methane oxidation rate (μmol CH_4_ h^-1^ g dry soil^-1^), m is the slope or decrease in methane concentration in the sample over time (ppmh^-1^), V _chamber_ is the volume of the gas chamber (l), W_sample_ is the weight of the sample (g), m_CH4_ the molar mass of methane (g mol^-1^) and μ the gravimetric water content (dimensionless). Results of all methane oxidation rate measurements are given in Supplementary information SI1.

### DNA Extraction

DNA was extracted from each microcosm at 11 time points throughout the experiment, using the Powersoil DNA isolation kit (Qiagen, Venlo, Netherlands), following the manufacturers protocol. This resulted in a total of 264 DNA samples. Quality and concentration of the DNA samples was determined spectrophotometrically (Nanodrop Technology, Wilmington, DE, United States). All samples had an OD_260_/OD_280_ ratio between 1.8 and 2, indicating good DNA quality.

### Microbial Community Composition – Sequencing of the 16S rRNA Gene

Microbial community composition in each sample was profiled based on diversity in the 16S rRNA genes, using barcode PCR, as described by Herbold et al. (2015) (**Table 1**). Briefly, 16S genes were PCR amplified using primers 515F and 806R with an attached head sequence (Caporaso et al., 2011). A second PCR on the initial product used a primer with the head sequence and a unique bar code, ensuring sequencing reads could be linked to a sample. Because for each sample there were already six experimental replicates, no technical PCR replicates were included.

**Table 1:** PCR programs used for preparation of 16S sequencing samples.

Step	Primer set	Primer sequence	PCR thermal profile
16S gene amplification	515F/806R + head sequence	5′ – GCTATGCGCGAGCTGC – GTGCCAGCMGCCGCGGTAA – 3′ (Head - 515F) 5′ – GCTATGCGCGAGCTGC – GGACTACHVGGGTWTCTAAT – 3′ (Head – 806R)	95°C, 1 min; 53°C, 1 min; 72°C, 1 min (30 cycles)
Barcode attachment	Head/barcode primer	5′ – barcode – GCTATGCGCGAGCTGC – 3′	95°C, 30 s; 53°C, 30 s; 72°C, 45 s (10 cycles)

All PCR products were checked for PCR product on a 1% agarose gel, and purified using the QIAquick PCR Purification Kit (Qiagen, Venlo, Netherlands). PCR product concentration and quality was measured using a Fragment Analyzer (Advanced Analytical, Heidelberg, Germany) (SI3). Samples for which the correct PCR product was absent or contaminated were discarded. Finally, all samples for which sufficient PCR product was found (242) were pooled in equimolar quantities, and MiSeq sequenced at LCG genomics (Berlin, Germany), resulting in 20,000,000 paired-end reads (see SI2 for a list of sequenced samples).

Sequencing data were analyzed using the Mothur sequence analysis software (Schloss et al., 2009) (**Table 2**). Paired-end reads were assembled into contigs and separated by barcode. Contigs were trimmed according to q-scores of the reads, and screened for unexpected lengths. Screened contigs were aligned against the current newest (v128) Silva database for alignments and taxonomy (Quast et al., 2012). Sequences aligning to a different region of the 16S gene were deleted. After this, chimeras were detected and removed using UCHIME (Edgar et al., 2011). Remaining sequences were classified to generate OTU tables for every soil sample (95% sequence similarity cutoff). OTUs mapping to the same taxonomy were merged using an in-house python script. When referring to microbial species found in this analysis, we do not refer to previously characterized species but rather to these OTUs, most of which do not share 100% sequence similarity with previously described species.

**Table 2:** Screening steps of 16S sequencing data analysis.

Processing step	Total sequences	% Remaining
Raw data	20000000	100.00
Contig assembly and trimming according to q-scores	14141936	70.71
Length screening of contigs (250–300 bp)	13645887	68.23
Alignment to v4 section of 16S gene (Silva database)	9968857	49.84
Filtering out alignments to different regions	9844212	49.22
Chimera detection	7747642	38.74
Classification (remove non-classified and rare sequences)	7561217	37.81

### Microbial Abundance – qPCR Assays

Two quantitative PCR assays were performed on extracted DNA from all samples: EUBAC and MTOT, targeting the 16S rRNA gene of all bacteria and the *pmoA* gene, respectively (Kolb et al., 2003). Although the MTOT assay does not target all MOB (it does not quantify MOB that do not express *pmoA*), it targets most MOB that are active and predominant in the sampled soil, as MOB that do not express *pmoA* are mainly restricted to marine or acidic environments [see for instance (Dunfield et al., 2003; Vorobev et al., 2011)]. For the qPCR assays, all samples were diluted to a DNA concentration of 2 μg/ml. For the EUBAC assay, one reaction consists of 7.5 μl iTaq universal supermix (Bio-rad, Veenendaal, Netherlands), 1.5 μl Bovine Serum Albumin (5 mg ml^-1^; Invitrogen), 0.75 μl forward and reverse primer, 2 μl PCR grade water, and 2.5 μl DNA sample. For the MTOT assay, one reaction consists of 10 μl 2x sensiFAST SYBR (BIOLINE, Alphen aan den Rijn, Netherlands), 1 μl of forward and reverse primers, 5.5 μl PCR grade water, and 2.5 μl DNA sample. The qPCR assays were performed with primers and PCR thermal profiles as described in **Table 3**. All qPCR assays were performed using a Rotor-Gene Q real-time PCR cycler (Qiagen, Venlo, Netherlands). Quantification was performed using a calibration curve of DNA with known amounts of 16S rRNA gene or *pmoA* gene for the EUBAC and *pmoA*-total assay, respectively. A concentration range of 10^7^–10^2^ copies/reaction was used for the calibration curve. Amplification specificity was verified from melt curves.

**Table 3:** Primers and PCR programs for the qPCR assays used in the experiment.

Assay	Primer set	Primer sequence	PCR thermal profile
EUBAC	EUB338F/EUB518R (Fierer et al., 2005)	5′ – ACTCCTACG GGAGGCAGCAG – 3′ (338F) 5′ – ATTACCGCG GCTGCTGG – 3′ (518R)	95°C, 10 s; 53°C, 10 s; 72°C, 25 s (data acquiring) (40 cycles)
pmoA total	A189F/mmb661R (Costello and Lidstrom, 1999)	5′ – GGNGACTGGGACTTCTGG – 3′ (A189F) 5′ – CCGGMGCAACGTCYTTACC – 3′ (mmb661R)	95°C, 10 s; 58°C, 15 s; 72°C, 25 s, 82°C, 10 s (data acquiring) (45 cycles)

### Statistical Analyses

All statistical analyses were performed in R statistics (R Core Team, 2015). Comparisons of treatments within time points were done by performing analysis of variance using Bonferroni adjustment for multiple testing. Comparisons between different time points within one treatment were made by performing paired-end *t*-tests using Bonferroni adjustment for multiple testing. Before testing, normality of the data and equality of variance was checked using the corresponding diagnostic plots. The 50 most abundant bacterial species in soil samples were tested for correlation with methane oxidation rate by calculating the Pearson correlation coefficient between the normalized abundance of the species and the methane oxidation rate over time. Methanotrophs and non-methanotrophs were included in this analysis, because non-methanotrophs may have a role in modulating methane oxidation rates. Then, for each species a *t*-test was performed to test whether the average correlation coefficient across the six replicates was different from zero. Again, *p*-values were adjusted for multiple testing using Bonferroni adjustment. Plots were generated in Python, using the Matplotlib package (Hunter, 2007), except for the PCA plot, which was created in R, using the ggbiplot package (Vu, 2011), after Hellinger transformation of the data.

## Author Contributions

AV and PB conceived the design of the work. HvK performed the analyses with input from AV, PB, and MM-K and wrote the initial manuscript. AV, PB, AH, MM-F, and HvK critically revised and approved the manuscript. All authors are accountable for all aspects of the work.

## Conflict of Interest Statement

The authors declare that the research was conducted in the absence of any commercial or financial relationships that could be construed as a potential conflict of interest.
