# Supplementary material for: Resistance and Recovery of Methane-Oxidizing Communities Depends on Stress Regime and History; A Microcosm Study
Source: Front Microbiol. 2018 Jul 31;9:1714. doi: 10.3389/fmicb.2018.01714 (PMC6080070; doi:10.3389/fmicb.2018.01714)
Supplement: Supplementary file 1 [file Data_Sheet_1.DOCX]

Supplementary Material

**Resistance and Recovery of Methane Oxidizing Communities depends on Stress Regime and History.**

Henri van Kruistum^1^, Paul L. E. Bodelier^1^, Adrian Ho^2^, Marion Meima-Franke^1^, Annelies J. Veraart^1,3*^

*** Correspondence:** Annelies Veraart: [a.veraart@science.ru.nl](mailto:a.veraart@science.ru.nl)

# Supplementary Figures and Tables

## SI 1 statistical tests

P-values for paired-end t-tests comparing methane oxidation rate at t­_0_ with time points after each perturbation within the same treatment (table S1.1), as well as p-values for ANOVA tests testing for a treatment effect in 16S qPCR data within the same time point (table S1.2). P-values were adjusted for multiple testing using Bonferroni adjustment.

Table S1.1. P-values of pairwise t-tests comparing methane oxidation rate between time points within treatments. Significance codes: 0 ‘***’ 0.001 ‘**’ 0.01 ‘*’ 0.05

| **Drought effect:** | **p.adj** | **significance** |
| --- | --- | --- |
|  |  |  |
| t1D vs t2D | 0.0016 | ** |
| t1D vs t4D | 0.0008 | *** |
| t1D vs t6D | 0.0033 | ** |
| t1D vs t8D | 0.0168 | * |
|  |  |  |
| **ammonium effect:** |  |  |
| t1A vs t8A | 0.0024 | ** |
| t1AD vs t8AD | 0.0014 | ** |

Table S1.2. P-values of pairwise t-tests comparing 16S copy numbers between treatments within the same time point. C = control, D = drought, A = ammonium, AD = ammonium+drought

| ***t1*** | **A** | **AD** | **C** |  | ***t2*** | **A** | **AD** | **C** |
| --- | --- | --- | --- | --- | --- | --- | --- | --- |
| **AD** | 1 | NA | NA |  | **AD** | 1 | NA | NA |
| **C** | 1 | 1 | NA |  | **C** | 1 | 1 | NA |
| **D** | 1 | 1 | 1 |  | **D** | 1 | 1 | 1 |
|  |  |  |  |  |  |  |  |  |
| ***t3*** | **A** | **AD** | **C** |  | ***t4*** | **A** | **AD** | **C** |
| **AD** | 0,810 | NA | NA |  | **AD** | 0,995 | NA | NA |
| **C** | 1 | 1 | NA |  | **C** | 1 | 0,522 | NA |
| **D** | 0,282 | 1 | 0,367 |  | **D** | 1 | 0,787 | 1 |
|  |  |  |  |  |  |  |  |  |
| ***t5*** | **A** | **AD** | **C** |  | ***t6*** | **A** | **AD** | **C** |
| **AD** | 0,797 | NA | NA |  | **AD** | 1 | NA | NA |
| **C** | 0,465 | 1 | NA |  | **C** | 1 | 0,96 | NA |
| **D** | 1 | 1 | 1 |  | **D** | 1 | 1 | 0,986 |
|  |  |  |  |  |  |  |  |  |
| ***t7*** | **A** | **AD** | **C** |  | ***t8*** | **A** | **AD** | **C** |
| **AD** | 1 | NA | NA |  | **AD** | 1 | NA | NA |
| **C** | 1 | 1 | NA |  | **C** | 1 | 1 | NA |
| **D** | 0,078 | 0,358 | 0,654 |  | **D** | 1 | 1 | 1 |
|  |  |  |  |  |  |  |  |  |
| ***t9*** | **A** | **AD** | **C** |  | ***t10*** | **A** | **AD** | **C** |
| **AD** | 1 | NA | NA |  | **AD** | 1 | NA | NA |
| **C** | 0,480 | 0,559 | NA |  | **C** | 1 | 1 | NA |
| **D** | 0,015 | 1 | 0,005 |  | **D** | 1 | 1 | 0,784 |
|  |  |  |  |  |  |  |  |  |
| ***t11*** | **A** | **AD** | **C** |  |  |  |  |  |
| **AD** | 1 | NA | NA |  |  |  |  |  |
| **C** | 1 | 1 | NA |  |  |  |  |  |
| **D** | 0.070 | 0,858 | 0,010 |  |  |  |  |  |

Table S1.3. P-values of pairwise t-tests comparing *pmoA* copy numbers between treatments within the same time point. C = control, D = drought, A = ammonium, AD = ammonium+drought

| ***t1*** | **A** | **AD** | **C** |  | ***t2*** | **A** | **AD** | **C** |
| --- | --- | --- | --- | --- | --- | --- | --- | --- |
| **AD** | 0,340 | NA | NA |  | **AD** | 1 | NA | NA |
| **C** | 0,834 | 1 | NA |  | **C** | 1 | 1 | NA |
| **D** | 0,407 | 1 | 1 |  | **D** | 1 | 1 | 1 |
|  |  |  |  |  |  |  |  |  |
| ***t3*** | **A** | **AD** | **C** |  | ***t4*** | **A** | **AD** | **C** |
| **AD** | 0,876 | NA | NA |  | **AD** | 1 | NA | NA |
| **C** | 1 | 1 | NA |  | **C** | 1 | 1 | NA |
| **D** | 0,719 | 1 | 1 |  | **D** | 0,638 | 1 | 1 |
|  |  |  |  |  |  |  |  |  |
| ***t5*** | **A** | **AD** | **C** |  | ***t6*** | **A** | **AD** | **C** |
| **AD** | 1 | NA | NA |  | **AD** | 0,136 | NA | NA |
| **C** | 1 | 0,389 | NA |  | **C** | 0,595 | 0,027 | NA |
| **D** | 0,428 | 0,790 | 0,150 |  | **D** | 0,144 | 1 | 0,027 |
|  |  |  |  |  |  |  |  |  |
| ***t7*** | **A** | **AD** | **C** |  | ***t8*** | **A** | **AD** | **C** |
| **AD** | 1 | NA | NA |  | **AD** | 0,913 | NA | NA |
| **C** | 1 | 0,090 | NA |  | **C** | 1 | 0,444 | NA |
| **D** | 1 | 0,049 | 0,784 |  | **D** | 0,691 | 1 | 0,372 |
|  |  |  |  |  |  |  |  |  |
| ***t9*** | **A** | **AD** | **C** |  | ***t10*** | **A** | **AD** | **C** |
| **AD** | 1 | NA | NA |  | **AD** | 1 | NA | NA |
| **C** | 0,436 | 0,148 | NA |  | **C** | 1 | 1 | NA |
| **D** | 0,017 | 0,159 | 0,039 |  | **D** | 0,892 | 0,876 | 0,152 |
|  |  |  |  |  |  |  |  |  |
| ***t11*** | **A** | **AD** | **C** |  |  |  |  |  |
| **AD** | 1 | NA | NA |  |  |  |  |  |
| **C** | 1 | 0,659 | NA |  |  |  |  |  |
| **D** | 0,057 | 0,661 | 0,009 |  |  |  |  |  |

## SI5 Fraction of methanotrophs according to qPCR assays and 16S sequencing data

A measure for the ratio between MOB and total amount of bacteria could be obtained in two ways, given the data obtained in this study: as the amount of reads mapping to the 16S gene of MOB compared to the total amount of reads per sample (figure S1A), or as the ratio between the *pmo*A and EUBAC qPCR assays, targeting the *pmoA* and 16S gene, respectively (figure S1B). Both methods show the same trend (samples subject to drought have a lower MOB fraction compared to the other samples). However, the sequencing method consistently estimates this fraction 2 to 3 times higher compared to the qPCR method. This could be due to a few reasons:

- 16S copy number in proteobacteria is often higher than the *pmoA* copy number, leading to an underestimation of the MOB/total bacteria ratio (Stolyar et al., 1999;Crosby and Criddle, 2003).
- The *pmoA* assay does not target all MOB, for instance *Verrucomicrobial* *pmoA* is not amplified.

Because of this, we chose to use the 16S sequencing method for our calculation of MOB fraction in soil. This method still has its own bias due to varying 16S copy number across groups of bacteria, but we believe it is a closer estimate than the qPCR method.


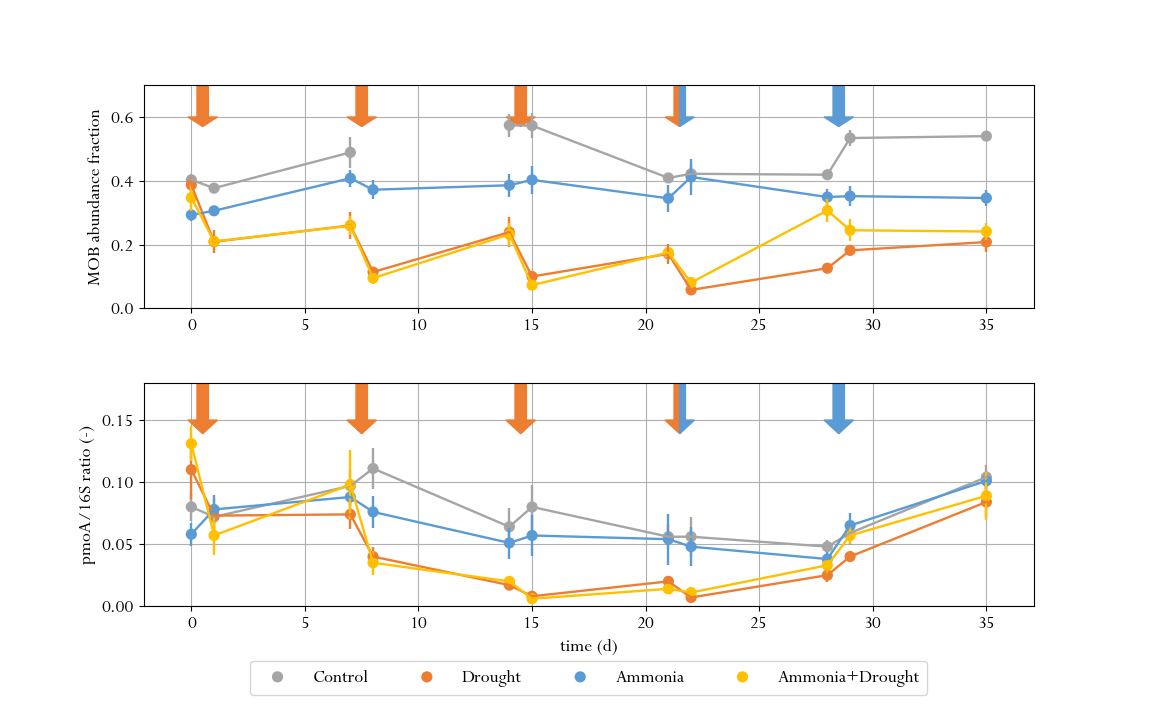


**A**

**B**

Figure S1. MOB/total bacteria ratio as calculated from 16S sequencing reads (S1A) and pmoA/16S qPCR assays (S1B). orange arrows indicate desiccation, blue arrows indicate ammonium addition.

## Batch effect

Unfortunately, when setting up the control microcosms the soil was not properly mixed, leading to a different initial starting community composition in the t_0_ control and treatment microcosms, whereas communities in the treatment microcosms were similar (see methods, section 4.1). In the next figures, methane oxidation rate (fig. S2) and community (fig. S3-4) data including the control group is shown. Additionally, a PcoA plot of community data at t_0_ is shown, visualizing the difference in microbial community of the control group at t_0_ (fig. S5).


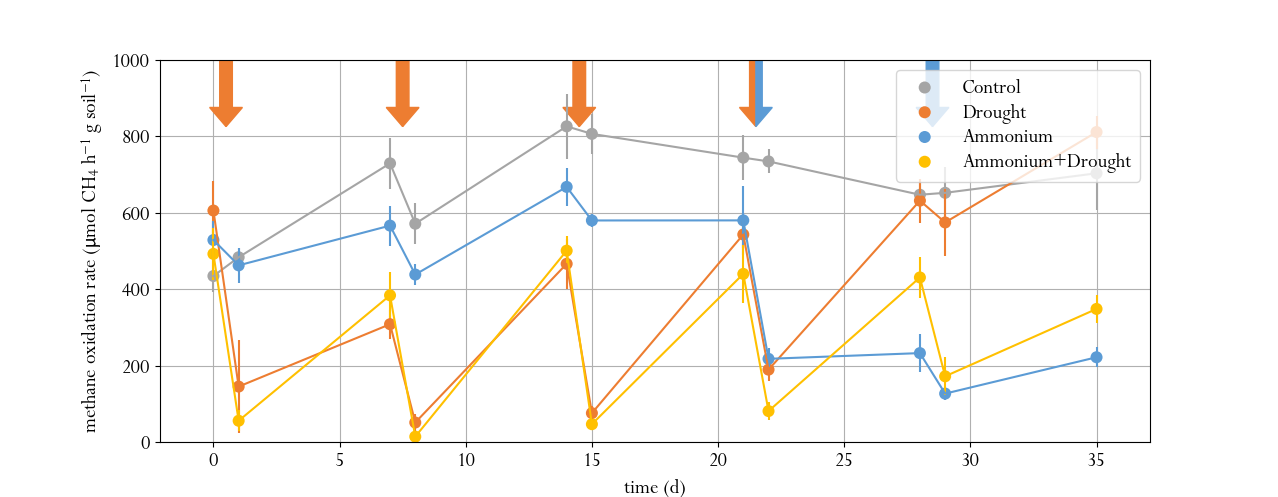


**Figure S2**. Methane oxidation rate in soil microcosms exposed to different perturbation regimes. Orange arrows indicate desiccation, blue arrows indicate ammonium addition to the corrensponding treatments. Error bars represent standard error of the mean.


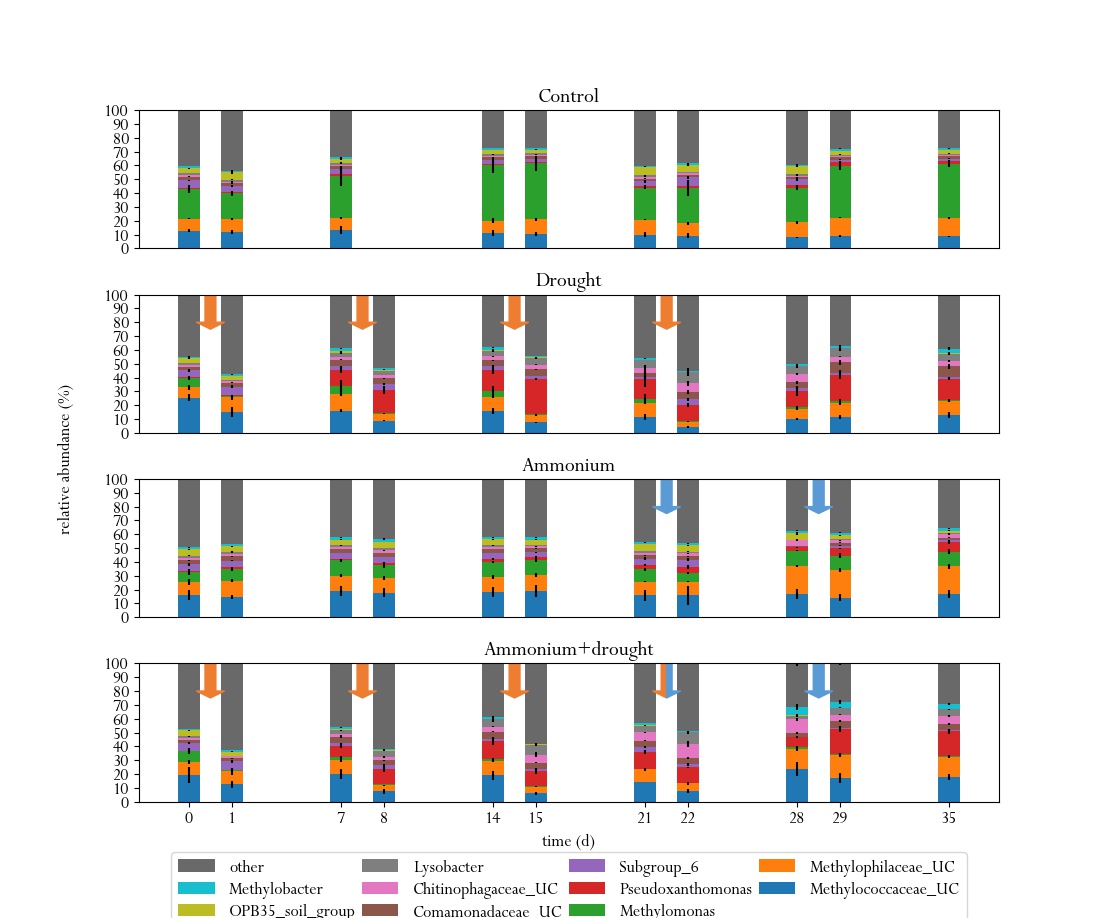


**Figure S3**. Relative abundances of ten most abundant bacterial genera in the four different treatments, before and after each perturbation. Orange arrows indicate desiccation events, blue arrows indicate ammonium addition to the corrensponding treatments.


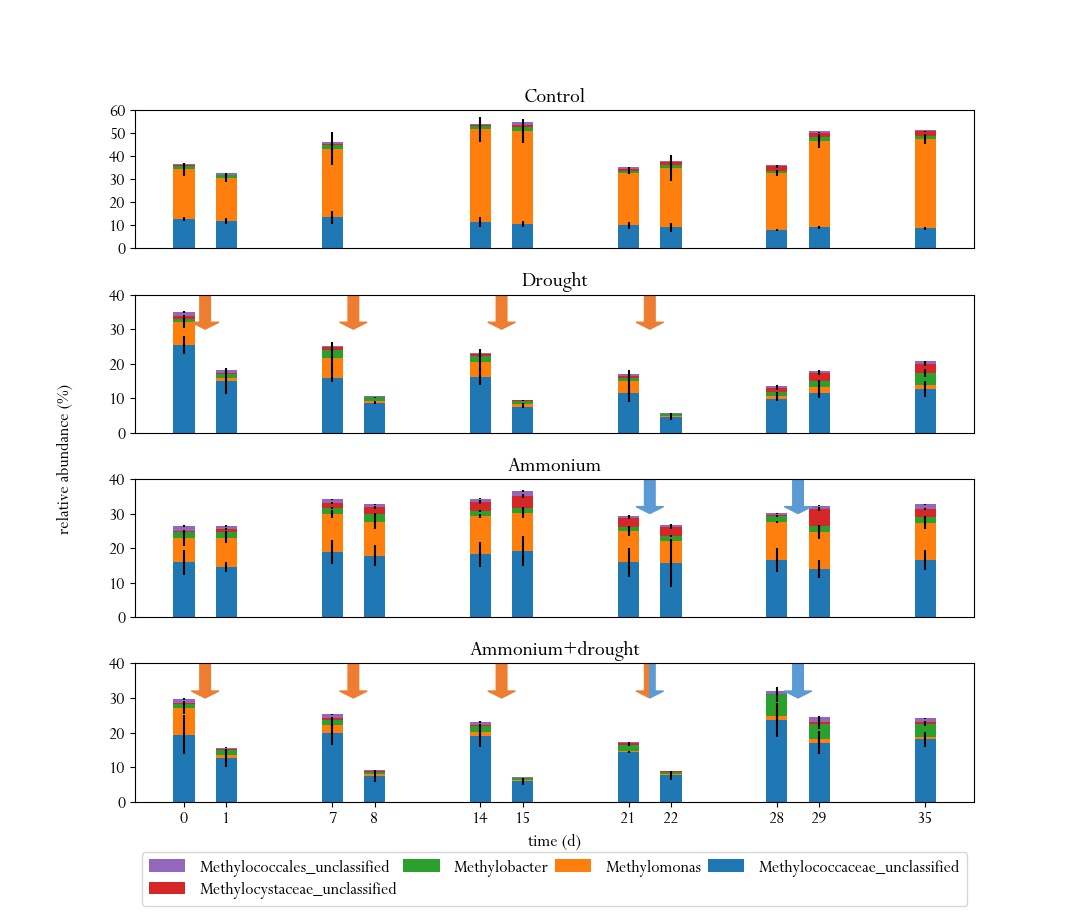


**Figure S4.** Relative abundances of MOB in the three different treatments, before and after each perturbation. Orange arrows indicate desiccation events, blue arrows indicate ammonium addition to the corrensponding treatments.


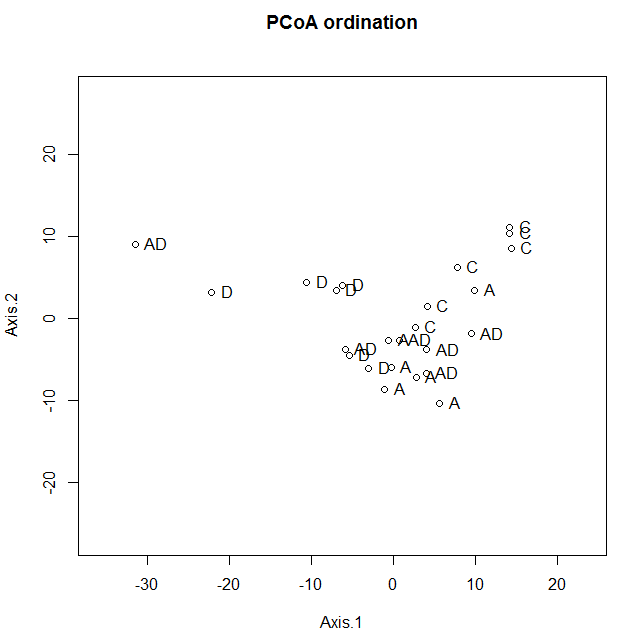


**Figure S5.** PcoA plot indicating differences in microbial community between samples at t_0_. Letters indicate treatment: C = control, D = drought, A = ammonium, AD = ammonium + drought

## References

Crosby, L.D., and Criddle, C.S. (2003). Understanding bias in microbial community analysis techniques due to rrn operon copy number heterogeneity. *Biotechniques* 34**,** 790-803.

Stolyar, S., Costello, A.M., Peeples, T.L., and Lidstrom, M.E. (1999). Role of multiple gene copies in particulate methane monooxygenase activity in the methane-oxidizing bacterium Methylococcus capsulatus Bath. *Microbiology* 145**,** 1235-1244.
